# Role of Neurotrophins in the Development and Treatment of Neurodegenerative Diseases: A Systematic Review

**DOI:** 10.7759/cureus.74048

**Published:** 2024-11-19

**Authors:** Sadia Mansoor, Anushka Jindal, Nana Yaw Afriyie Badu, Chiko Katiki, V Jaswitha S Ponnapalli, Kesha J Desai, Sondos T Nassar

**Affiliations:** 1 Medicine, Dow University of Health Sciences, Karachi, PAK; 2 Surgery, University Hospital Sussex, Worthing, GBR; 3 Surgery, University of Ghana, Accra, GHA; 4 Emergency Medicine, American International Medical School, Santa Rosa, USA; 5 Psychiatry, California Institute of Behavioral Neurosciences and Psychology, Fairfield, USA; 6 Medicine, Medical College Baroda, Vadodara, IND; 7 Medicine and Surgery, Jordan University of Science and Technology, Amman, JOR

**Keywords:** dementia, neurodegeneration, neurotrophic factors, neurotrophins, progressive neuron loss

## Abstract

A considerable amount of morbidity and disability are caused by a wide variety of neurological illnesses together referred to as neurodegenerative diseases. Among them, Alzheimer’s and Parkinson’s diseases are the most prevalent and have been thoroughly studied. The development of intervention techniques that focus on the unfavorable elements of these diseases, particularly those that could help halt their course, has become increasingly important. This study aims to explain the most current findings about the function of neurotrophins, the signaling pathways they follow in neurodegenerative illnesses, and their possible therapeutic applications. The Preferred Reporting Items for Systematic Reviews and Meta-Analyses (PRISMA) 2020 criteria served as the foundation for this systematic review. In April 2024, a thorough search was conducted through the Cochrane Library, Google Scholar, PubMed, PubMed Central, and ScienceDirect databases. The predetermined criteria used to choose the research were the English language, narrative and systematic reviews, observational studies, and randomized and non-randomized clinical trials published within the last ten years. Subsequently, each study type-specific quality assessment was conducted utilizing the available assessment method. Of the 3,322 studies found during the first search, 15 were ultimately chosen for inclusion in the final selection. One cohort, one non-randomized clinical trial, one randomized clinical trial, three meta-analyses and systematic reviews, and nine narrative reviews were included. This review has explained in detail the current understanding of how neurotrophins play an essential role in neuroplasticity and neurogenesis, as well as their complex downstream signaling that leads to the process of neurodegeneration. Our study has also highlighted previous studies showing the efficacy of neurotrophins in clinical trials, but the data is limited; more preclinical and clinical studies are needed in this regard. These have also drawn our attention to future clinical trials that will address the challenges faced in their delivery and associated complications. Altogether, neurotrophins could serve as promising targets for therapeutic intervention that could stop or even reverse the development of neuropathology associated with neurodegenerative illnesses.

## Introduction and background

Neurodegenerative diseases are a diverse range of neurological conditions that cause gradual loss of neurons in the central or peripheral nervous systems, impacting millions of individuals globally [1]. Alzheimer's disease (AD) is the most common neurological condition, which affects 11% of people over 65 and over half of those 85 and older [2]. Current estimates indicate that neurological diseases account for a significant portion of morbidity and disability and that the percentage of new cases of these diseases will likely increase [3]. Neurodegenerative diseases frequently cause gradual behavioral, functional, and cognitive alterations that show up as impaired motor and mental performance [4]. Cognitive and behavioral dysfunctions, as well as a significant reduction in life quality, are expected outcomes of neurodegenerative disorders, which are primarily characterized by the progressive death of neurons and a deterioration in neuronal function [5]. Numerous potential processes of neurodegeneration have been proposed based on transgenic models of illnesses and toxicological studies [6]. Common characteristics and methods include the build-up of intrinsically disordered proteins in aggregates, the role of oxidative stress and mitochondrial failure, and neuroinflammation [6]. Though having a deep understanding of the pathophysiology of these illnesses, the precise mechanisms and causes of neurodegeneration are still unknown [6]. Instead of using therapy to address the disease's symptoms, there is a need to impede the targeted search for new, effective neuroprotectors and therapeutic approaches that could stop or at least delay the advancement of the disease [6]. Examining the molecular processes behind the emergence of neurodegenerative disorders and future perspectives on neuronal protection becomes essential from these perspectives to promote the creation of efficient therapeutic disease-modifying strategies [6].

A unique strategy for preserving and repairing neuronal function in the central nervous system is using neurotrophins as therapeutic agents, which can promote neuronal sprouting, shield injured and diseased neurons from dying, and improve the metabolism and function of neurons [7]. Neurotrophins are proteins with similar sequences and structures derived from a common ancestral gene. These proteins include nerve growth factor (NGF), brain-derived neurotrophic factor (BDNF), neurotrophin-3 (NT3), and neurotrophin-4/5 (NT4/5). BDNF is one of the neurotrophins studied in detail [4]. Neurotrophins, like many other growth factors, are produced as pro-forms, which are then converted to mature neurotrophins by cleaving the N-terminal prodomain. Mature neurotrophins, such as neurotrophins (NT) 3-6, BDNF, and NGF, bind to the appropriate family of receptor tyrosine kinases known as tropomyosin receptor kinase (Trk), having three subtypes, TrkA, TrkB, and TrkC. The complicated nature of neurotrophic receptor signaling probably explains the variety of neuronal and non-neuronal activities of neurotrophins [8]. 

Because of a void in the body of current literature regarding the exact role of neurotrophins in the development of neurodegeneration, this review aims to explain how neurotrophins contribute to the pathophysiology of neurodegenerative diseases. We also present recent updates on the role of neurotrophins and their downstream signaling pathways in these diseases, including Alzheimer’s and Parkinson’s disease. In addition, we will also review the therapeutic potential of neurotrophins in treating neurodegenerative diseases.

## Review

Methods

This study is formulated on PRISMA 2020 guidelines [9].

Eligibility Criteria

This review question was meticulously crafted based on the PIO criteria encompassing participants, intervention, and outcome. Our study focuses on patients afflicted with neurodegenerative diseases such as Alzheimer’s and Parkinson’s disease. The intervention is neurotrophic factors, and the result is the role of neurotrophins in the development of neurodegenerative diseases and the efficacy of their treatment.

*Inclusion Criteria*

Our study included free full-text articles and papers published in English in the last 10 years, including narrative and systematic reviews, clinical trials, and observational studies.

Exclusion Criteria

Our study excluded papers and articles published before 2014 in languages other than English language and animal studies. 

Databases and Search Strategy

Five databases-PubMed, PubMed Central (PMC), Google Scholar, Cochrane Library, and ScienceDirect, were used in a systematic search. Neurotrophins, nerve growth factors, dementia, progressive neuron loss, and neurotrophic factors were the critical terms employed. The Medical Subject Heading (MESH) approach was employed to extract the data from PubMed. EndNote was used to organize and alphabetize all the references, and both Endnote and human labor were used to eliminate duplicates. Subsequently, the data underwent screening using titles and abstracts to remove research that was deemed unnecessary. The full-text articles were retrieved. Table 1 lists the databases and search strategies in detail.

**Table 1:** Search strategies across different databases

Databases	Keywords	Search Strategy	No of articles before filters	Filters	Search Results
PubMed	Neurodegenerative diseases OR progressive neuron loss OR dementia AND neurotrophins OR nerve growth factors OR neurotrophic factors	Neurodegenerative diseases OR progressive neuron loss OR dementia OR ( "Neurodegenerative Diseases/chemically induced"[Majr] OR "Neurodegenerative Diseases/classification"[Majr] OR "Neurodegenerative Diseases/drug therapy"[Majr] OR "Neurodegenerative Diseases/etiology"[Majr] OR "Neurodegenerative Diseases/genetics"[Majr] OR "Neurodegenerative Diseases/immunology"[Majr] OR "Neurodegenerative Diseases/metabolism"[Majr] OR "Neurodegenerative Diseases/pathology"[Majr] OR "Neurodegenerative Diseases/physiopathology"[Majr] OR "Neurodegenerative Diseases/therapy"[Majr] ) AND Growth factors OR neurotrophins OR nerve growth factors OR ( "Nerve Growth Factors/adverse effects"[Majr] OR "Nerve Growth Factors/analysis"[Majr] OR "Nerve Growth Factors/biosynthesis"[Majr] OR "Nerve Growth Factors/chemical synthesis"[Majr] OR "Nerve Growth Factors/classification"[Majr] OR "Nerve Growth Factors/drug effects"[Majr] OR "Nerve Growth Factors/pharmacokinetics"[Majr] OR "Nerve Growth Factors/pharmacology"[Majr] OR "Nerve Growth Factors/physiology"[Majr] OR "Nerve Growth Factors/therapeutic use"[Majr]	16,529	Papers from 2014 – 2024 Free full text articles English Human Studies	947
Cochrane Library	-	Neurodegenerative diseases OR progressive neuron loss OR dementia AND neurotrophins OR nerve growth factors OR Neurotrophic factor	176	Papers published from 2014-2024 English language	129
Google Scholar	-	"Neurodegenerative diseases" OR "progressive neuron loss" OR "dementia" AND "neurotrophins" OR "nerve growth factors" OR “neurotrophic factor”	6,589	Papers from 2014-2024	1,045
ScienceDirect	-	Neurodegenerative diseases AND Neurotrophins	7,025	Papers from 2014-2024 English Review articles	320
PubMed Central	-	Neurodegenerative diseases OR progressive neuron loss OR dementia AND neurotrophins OR nerve growth factors OR Neurotrophic factor	4,2085	Papers published in the last ten years Open access	881

Results

**Figure 1:**
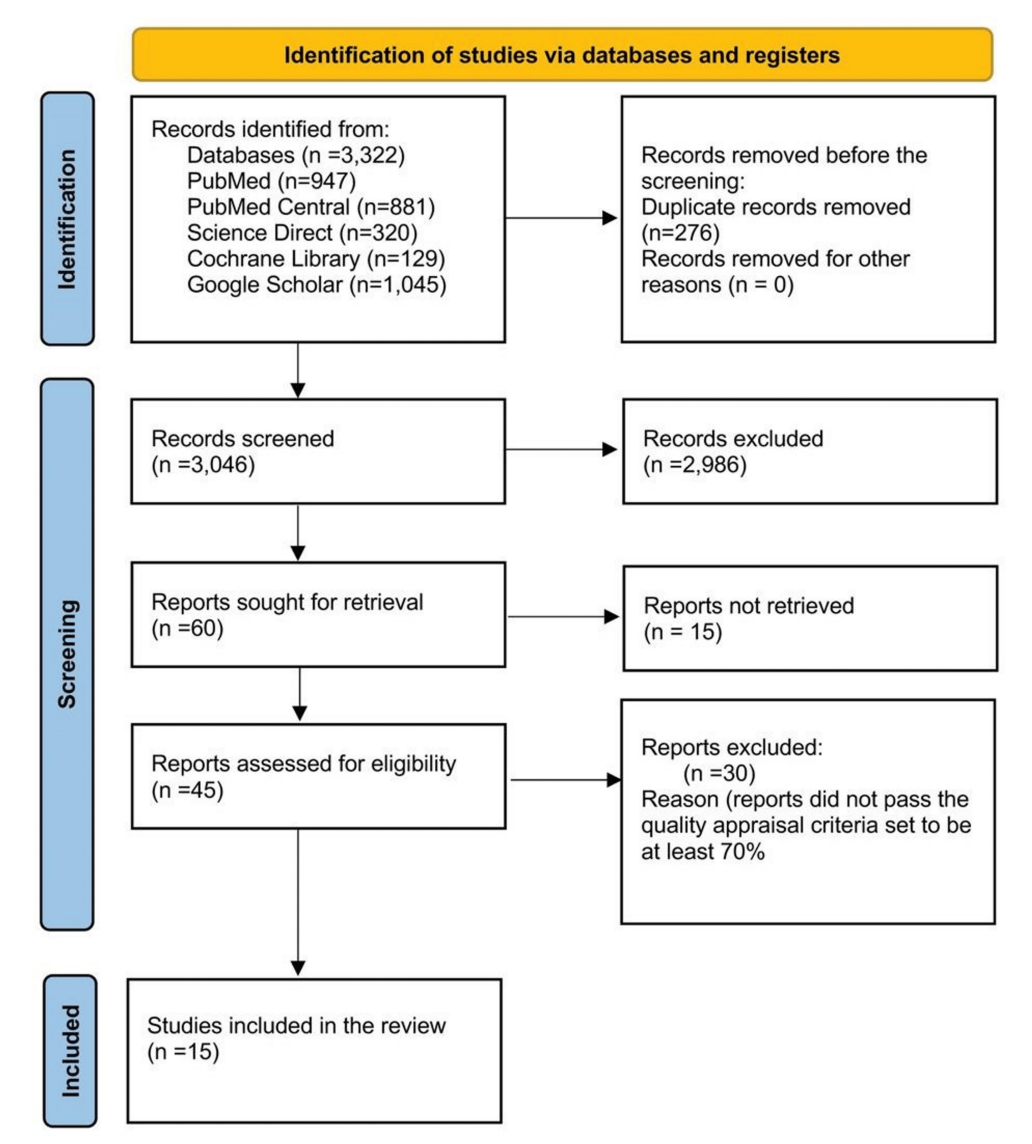
PRISMA 2020 flow chart of the search strategy of studies PRISMA, Preferred Reporting Items for Systematic Reviews and Meta-Analysis [9].

The database search began with 3,322 potentially pertinent titles and 3,046 records remaining after eliminating duplicates. Next, 45 articles were left for retrieval after 2,986 articles were eliminated throughout screening the titles and abstracts of the records by the (population, intervention, control group, outcome) aspects of the review. Ultimately, the first author evaluated the quality of the retrieved reports, and the second author reviewed and approved the results. This process produced the fifteen studies that received a score of greater than 70% via quality assessment tools and were included in the study. Nine narrative reviews, three meta-analyses, one cohort, one randomized, and one non-randomized clinical trial were included in this set. Figure 1 explains a flow chart of the study design and screening procedure.

The entire papers that were retrieved underwent quality assessment and risk of bias assessment using tools specific to the kind of study: Nine studies met the requirements to be accepted by the Scale for the Assessment of Narrative Review Articles 2 (SANRA 2) for narrative reviews; one study met the eligibility requirements for the Cochrane Collaboration Risk of Bias Tool (CCRBT) for randomized clinical trial with a score of greater than 70%; three papers pass the quality appraisal for Systematic Reviews and Meta-analyses using the Assessment of Multiple Systematic Reviews 2 (AMSTAR 2). Cohort studies and non-randomized clinical trials are rated with a quality appraisal score of at least 70% to be accepted according to the Newcastle Ottawa Scale (NOS). The requirements for each evaluation tool are detailed in Table 2 below.

**Table 2:** Description of Quality assessment tools SANRA 2, Scale for the Assessment of Narrative Review Articles 2; NOS, Newcastle Ottawa Scale; CCRBT, Cochrane Collaboration Risk of Bias Tool; AMSTAR 2, Assessment of Multiple Systematic Reviews 2; RCTs, Randomized controlled trials; PICO, population, intervention, control group, outcome

Quality assessment tool	Type of study	Items and their characteristics	Total Score	Accepted score (> 70 %)	No of accepted studies
SANRA 2 [10]	Narrative review	Six points: (1) the article's significance for the readership is justified (2) the question's specific purpose is described (3) the literature search is described; (4) references are made (5) scientific reasoning is applied; and (6) the data is presented in an appropriate manner. rated as 0, 1, and 2.	12	9	9
AMSTAR 2 [11]	Systematic review, Meta-analysis	sixteen items (1) Did the PICO component figure in the research question and inclusion criteria? (2) Was it specifically stated in the review report that the review techniques had been decided upon before the review was carried out? (3) Did the authors of the review provide an explanation for the study designs they chose to include in the review? (4) Did the review writers employ a thorough approach for searching the literature? (5) Were studies selected by the review authors in duplicate? (6) Were data extraction tasks completed in duplicate by the review authors? (7) Were the exclusions justified by the review authors? (8) Did the review writers provide a detailed description of the included studies? (9) Did the review writers evaluate the likelihood of bias in the included studies using a suitable tool? (10) Did the review authors provide an explanation of how the studies that were part of the review were funded? (11) Did the review employ suitable techniques for combining the results statistically? Did the review writers evaluate the possible influence of individual study bias on the meta-analysis results, if a meta-analysis was conducted? (13) When evaluating the review's findings, did the review writers take the possibility of bias in individual studies into consideration? (14) Did the review authors describe any heterogeneity they saw in the review's findings? (15) If quantitative synthesis is used, did the review writers sufficiently examine publication bias and how it affects the study's findings? (16) Were there any possible conflicts of interest disclosed by the review authors? Scored as YES or NO, Partial YES was considered a point.	16	12	3
CCRBT [12]	RCTs	Seven items: random sequence generation, allocation concealment, selective outcome reporting, other bias, blinding of participants and personnel, blinding of outcome assessment, incomplete outcome data Bias assessed as LOW RISK, HIGH RISK or UNCLEAR	7	5	1
NOS [13]	Cohort, Nonrandomized clinical trials	Eight items: (1) exposure cohort representation; (2) non-exposure cohort selection; (3) exposure determination; (4) evidence that the desired outcome was absent at study initiation; and (5) cohort comparability based on design and analysis.; (6)Evaluation of the result ; (7)follow-up duration sufficient for results to materialize ; (8) and sufficiency of cohort follow-up. Each category receives one point, with category five receiving a maximum of two points. Scored as YES, NO, and NOT APPLICABLE	9	7	2

The tables that follow explain the summary of the risk bias assessment of each study type using specific quality assessment tools. Table 3 below shows the summarized results of nine narrative reviews and describes the scoring on each item of Scale for the Assessment of Narrative Review Articles 2 (SANRA 2).

**Table 3:** Summary of quality assessment of narrative reviews

Studies	Significance for Readership	Research Question	Literature Search	Referencing	Scientific reasoning	Data Presentation	Total Score
G. Nordvall et al. 2022 [8]	2	2	2	2	2	2	12
N. Ali et al. 2024 [14]	2	2	1	2	2	2	11
T. Shen et al. 2017 [15]	2	2	1	2	1	2	10
L. Colucci-D'Amato et al. 2020 [16]	2	2	2	2	1	2	11
P. Chmielarz and M. Saarma 2020 [18]	2	2	2	2	2	1	11
N. Baazaoui and I. Khalid 2022 [19]	2	2	1	2	1	2	10
S. Cade et al. 2021 [20]	2	2	2	1	1	2	10
K. Delgado-Minjares et al. 2021 [25]	2	2	1	2	1	1	9
K. Azman et al. 2022 [4]	2	2	2	2	1	2	11

Table 4 below explains the scoring on each Assessment of Multiple Systematic Reviews 2 (AMSTAR 2) item for the included three systematic reviews and meta-analyses.

**Table 4:** Summary of quality assessment of systematic reviews and meta-analysis

Studies	Item 1	Item 2	Item 3	Item 4	Item 5	Item 6	Item 7	Item 8	Item 9	Item 10	Item 11	Item 12	Item 13	Item 14	Item 15	Item 16	Total Score
T. K. Ng et al. [17]	YES	YES	YES	No	YES	YES	NO	YES	YES	No	YES	YES	YES	YES	YES	NO	12
L. Jiang et al. [24]	YES	NO	YES	YES	YES	YES	YES	YES	PARTIAL YES	YES	YES	NO	YES	YES	YES	YES	14
Z. Chen et al. [26]	YES	YES	YES	PARTIAL YES	YES	YES	YES	YES	YES	YES	YES	NO	YES	YES	YES	YES	15

In our study, we included one non-randomized clinical trial and one longitudinal cohort study; both of the studies were assessed using the Newcastle Ottawa Scale, and their results are summarized below in Table 5

**Table 5:** Summary of studies assessed through NOS NOS, Newcastle Ottawa Scale

Studies	Item 1	Item 2	Item 3	Item 4	Item 5	Item 6	Item 7	Item 8	Results
M. Tuszynski et al. 2015 [21]	YES	YES	YES	YES	YES	YES	N/A	YES	PASS
S.D. Ginsberg, et al. 2019 [23]	YES	YES	YES	N/A	YES	YES	YES	YES	PASS

Our study has only one randomized clinical trial that scored more than 70% according to the Cochrane Collaboration Risk of Bias Tool CCRBT. Results are shown in Table 6.

**Table 6:** Results of quality assessment of randomized clinical trial

Studies	Selection Bias	Reporting Bias	Performance Bias	Detection Bias	Attrition Bias	Other bias	Results
M. S. Rafii et al. [22]	LOW-RISK	LOW-RISK	LOW RISK	UNCLEAR	LOW-RISK	LOW RISK	Included

Discussion

Biological Functions/Signaling Cascade of Neurotrophins

The brain and peripheral tissues express tiny proteins and growth factors called neurotrophins, which control many important aspects of neuronal functioning, such as neurogenesis, synaptic plasticity, and neuroprotection [14]. The four main structurally related protein types that make up the neurotrophic family are neurotrophin-3 (NT-3), neurotrophin-4 (NT-4), brain-derived neurotrophic factor (BDNF), and nerve growth factor (NGF) [15]. Due to variations in the receptor and accessory protein expression patterns, they have comparable actions on neurons but at different sites [14]. One of the neurotrophins most widely distributed and researched in the mammalian brain is brain-derived neurotrophic factor [16]. Several neuronal subtypes, including spinal motor neurons, cholinergic neurons, dopaminergic neurons, sensory neurons, and retinal ganglion cells, have shown increased BDNF regulation [15]. The cortical, hippocampal, and basal forebrain regions essential for memory, learning, and higher cognitive function express the BDNF gene [16]. BDNF is crucial for the healthy growth, development, and plasticity of glutamatergic and GABAergic synapses (GABA, gamma-aminobutyric acid) [16]. It modifies neuronal differentiation by affecting serotonergic and dopaminergic neurotransmission [16]. BDNF stimulates synaptic development, increases neurogenesis and neurotransmission across synapses, and modifies synaptic plasticity [17]. The alteration of mature neuronal morphology, which includes synaptogenesis, an increase in spine density, and axonal and dendritic arborization and pruning, contributes to neuronal plasticity [16]. Numerous in vivo and in vitro investigations examined how BDNF affected plasticity. The activation of intracellular signaling cascades most likely brings about this neuroplastic effect [16]. Recently, BDNF has been thoroughly investigated as a crucial modulator of synaptic transmission and long-term potentiation in the hippocampus [16]. Hippocampal long-term potentiation (LTP), a cellular process thought to be involved in memory formation, is strongly and positively modulated by BDNF [8,17]. LTP, or long-term strengthening of synapses between brain cells, is essential for developing learning and memory [15]. In the hippocampus and other brain parts, BDNF plays a significant role in controlling synaptic transmission and long-term potentiation [16]. The TrkB receptor mediates the effects of BDNF on LTP [16]. Thus, neurotrophins are believed to act on both the pre-and post-synaptic compartments, particularly in the hippocampus, modulating synaptic efficacy either by altering the release of pre-synaptic transmitters or by raising the sensitivity of post-synaptic transmitters to cause a sustained increase in synaptic plasticity [16].

**Figure 2:**
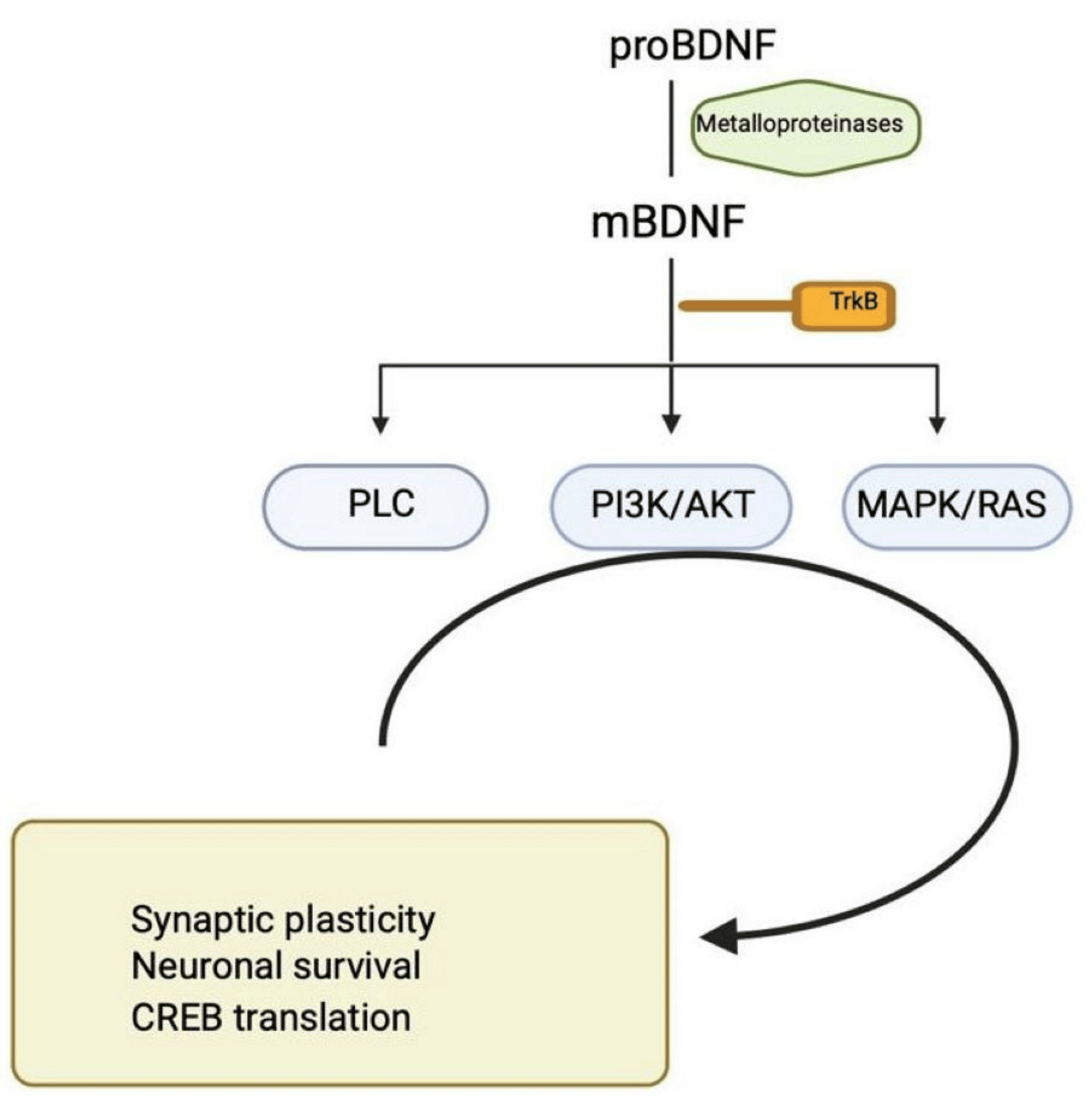
Mechanism of brain-derived neurotrophic factor via signaling pathways proBDNF, immature form of brain-derived neurotrophic factor; mBDNF, mature form of brain-derived neurotrophic factor; TrkB, tropomyosin receptor kinase B; PLC, phospholipase C pathway; PI3K/Akt, phosphatidylinositol-4,5-bisphosphate 3-kinase/protein kinase B; MAPK/RAS, mitogen-activated protein kinase/reticular activating system; CREB, cAMPresponse element-binding protein; cAMP, Ca+2 /calmodulin-dependent protein kinase. Created with BioRender.com by authors.

The 247 amino acid BDNF protein was discovered in 1982 and is a highly conserved protein that the endoplasmic reticulum produces and folds as preproBDNF [4,16]. As the region is translocated to the Golgi apparatus, its signal sequence is rapidly disrupted, making the isoform proBDNF [16]. The proBDNF is cleaved to provide the mature isoform mBDNF [16]. Plasmin and matrix metalloproteinases are needed for the extracellular cleavage of ProBDNF [4,16]. The ratio of proBDNF to mBDNF is determined by the distinct stages and regions of brain development [16]. ProBDNF may extensively regulate brain function and is more concentrated in the early postnatal period; adult-specific mBDNF is more common and essential for developed functions such as synaptic plasticity and neuroprotection [16]. Mature BDNF is released extracellularly and activates tropomyosin receptor kinase B (TrkB), which dimerizes and phosphorylates specific intracellular tyrosine residues [4,15]. Through its interaction with the TrkB receptor, mBDNF activates signaling cascades that support synaptic plasticity, neuronal survival, and cyclic adenosine monophosphate (cAMP) response element-binding protein (CREB) translation [18]. The mechanisms via which the mature form of BDNF carries out its neuroprotective effects are phospholipase C-γ pathways (PLCγ), phosphatidylinositol-4,5-bisphosphate 3-kinase (PI3K)/protein kinase B (Akt), and mitogen-activated protein kinase (MAPK) [18]. The phospholipase C pathway (PLC)-dependent pathway activates protein kinase C (PKC) and CAM kinase (Ca+2 /calmodulin-dependent protein kinase), which raises the amounts of Ca2+ ions and 1,2-diacylglycerol [8,16]. The PLC-dependent pathway, which stimulates protein kinase C (PKC) and calcium-calmodulin-dependent protein kinase (CAMK), is responsible for the increased synaptic plasticity [8,16]. This releases calcium ions from intracellular calcium storage and calcium-dependent signaling pathways [8,16]. The path associated with PI3K/Akt** **promotes survival and inhibits apoptosis by modulating synaptic plasticity that relies on the N-methyl-D-aspartate receptor [8,16]. The PI3K/Akt/mammalian target of rapamycin (mTOR)cascade promotes dendritic branching and growth by regulating protein synthesis and cytoskeleton development [8,16]. The PI3K/Akt pathway has pro-survival and anti-apoptotic effects, affecting N-methyl-D-aspartate (NMDA) receptor-dependent synaptic plasticity. Dendritic growth and branching, which regulates protein synthesis and cytoskeleton formation, are enhanced by the PI3K/Akt/mTOR cascade [8,16]. cAMP response element-binding protein (CREB is triggered by the mitogen-activated protein kinase (MAPK)/reticular activating system​​​​​​​ (RAS)-signaling cascade, which regulates protein synthesis throughout neuronal development [4,16]. The MAPK/RAS** **signaling cascade regulates protein synthesis during brain development and is also required to activate CREB [4,16]. The manufacture of cytoskeleton proteins like Arc and cypin and the development and branching of dendrites in hippocampal neurons depend on this pathway [4,16]. Figure 2 explains the different signaling pathways associated with BDNF. 

Role of Neurotrophins in Alzheimer's Disease

Roughly 70% of dementia cases worldwide, or approximately 35 million people, are affected by AD, the most common neurodegenerative disease [16]. Clinically, AD is marked by cognitive decline, behavioral abnormalities, and memory impairment [15]. Pathologically, the brain contains neurofibrillary tangles, beta-amyloid (Aβ), and hyperphosphorylated Tau protein plaques [15]. The primary area where neurons are lost in the hippocampal region increases as the disease progresses [18]. Memory loss has been directly associated with neurodegeneration in the cortical association areas [19]. Layer II of the entorhinal cortex exhibits the most significant neuronal loss in mild AD, setting it apart from that layer in older people without dementia [19]. Neurodegeneration in AD is a well-established process [20]. A person's annual decrease in brain mass can be as high as 0.5% in a normal-aged person and as much as 5% greater in an AD patient [20]. A neurotrophic imbalance associated with aging resulting from compromised activity-dependent processes may heighten the likelihood of synaptic malfunction, a precursory event in Alzheimer's disease [20]. The hippocampus of elderly rats and the cortex of aged mice exhibit decreased BDNF transcription, indicating that changes in its stimulus-driven expression occur as people age [20]. Age-related deficits in neurotrophic signaling are thought to increase the risk of AD in some people because aging-related poor activity-dependent mechanisms may make hippocampal neurons less responsive to stimulation for the transcription of Ca+/calmodulin-dependent protein kinases, which in turn causes a delayed or decreased increase in BDNF transcription [20]. Age-related decrease in the activity of both intracellular and extracellular proteases may also impede the commonly occurring rapid maturation of BDNF [20]. Overall, proBDNF rises compared to mBDNF, which raises the possibility of synaptic dysfunction [20].

It is well recognized that neurotrophins have an extrinsic modulator role in neurogenesis and aid in the maturation, migration, proliferation, and determination of the cell destiny of neural stem cells and neuro progenitor cells [19]. The control of adult hippocampal neurogenesis depends heavily on the BDNF/TrkB/CREB signaling pathways, which are also most likely to cause the neurogenesis deficit observed in AD [19]. One study showed that when patients with Alzheimer's disease are given nerve growth factor (NGF) therapy, they experience characteristic trophic responses, which include axonal sprouting [21]. These responses can last for ten years following gene transfer [21]. Further evidence for neurotrophic activity comes from a subset of patients tested for CREB activation and neuronal hypertrophy [21]. A non-randomized clinical trial using NGF gene therapy in AD patients was conducted by Tuszynski et al. in 2015 [21]. Using either ex vivo or in vivo gene transfer, 10 patients with early AD received NGF gene therapy [21]. Eight patients from the initial Phase 1 ex vivo experiment and two patients from a follow-up Phase 1 in vivo trial had their brains analyzed [21]. In the brain of AD patients, degenerating neurons react to NGF [21]. Axonal sprouting toward the NGF source was a trophic response seen in all patients in response to NGF [21]. In three patients who underwent unilateral gene transfer, cholinergic neuronal hypertrophy was observed on the NGF-treated side of the brain when comparing the treated and untreated sides (P>0.05) [21]. Two individuals with Adeno-associated viral vector serotype 2 (AAV2)-mediated NGF gene transfer had functional markers and activated cellular signaling [21]. Another study using AAV2-NGF was tested in a phase 2 randomized clinical trial by M. S. Rafii et al. to determine how it affected mild to moderate AD-associated dementia patients' cognitive decline [22]. Forty-nine individuals with mild to moderate AD were allocated randomly to receive either sham surgery or stereo tactically guided intracerebral injections of AAV2-NGF in a multicenter phase 2 trial [22]. At month 24, the cognitive subscale of the Alzheimer Disease Assessment Scale was evaluated for change from baseline [22]. For 24 months, AAV2-NGF was well tolerated and safe [22]. The Alzheimer Disease Assessment Scale-cognitive subscale, the primary outcome measure, showed no discernible difference between the treatment group and placebo (mean [SD] score, 14.52 vs 9.11, P =0.17) [22]. Delivery of AAV2-NGF was well tolerated; nevertheless, it had little effect on specific AD biomarkers or clinical outcomes [22]. Both PET scans and magnetic resonance imaging did not reveal any difference in the results when comparing treatment with placebo [22]. The outcomes of NGF gene therapy in clinical studies are displayed in Table 3.

**Table 7:** Effects of NGF gene therapy in clinical trials NGF, nerve growth factor; AAV2, adeno-associated viral vector (serotype 2); AD, Alzheimer's disease

Author	Study Type	Drug/Intervention Used	Conclusion
Tuszynski et al. [21]	Nonrandomized clinical trial	NGF gene therapy	Degenerating neurons in the AD brain respond to NGF. All patients exhibited a trophic response to NGF, in the form of axonal sprouting toward the NGF source
M S. Rafii et al. [22]	Randomized clinical trial	AAV2-NGF gene therapy	AAV2-NGF delivery was well-tolerated but did not affect clinical outcomes or selected AD biomarkers

One crucial mechanism in the growth and operation of neural networks is synaptic plasticity. It has been proposed that synaptic deficit precedes neurodegeneration, which is followed by tau and amyloid diseases and cognitive impairment in the very early stages of AD [19]. Dementia severity is closely correlated with synaptic loss [19]. A quantitative assessment of AD brains conducted 2-4 years after the disease's clinical diagnosis revealed a 25-35% decrease in synapses per neuron in the frontal and temporal cortices [19]. This loss is most severe in the hippocampal region, reaching 44-55% [19]. A synapse loss occurs in a break in the link between the innervating and target neurons, and synaptic transport dysfunction affects the retrograde transfer of neurotrophic substances [16]. These events are considered early in the pathophysiology of AD, which in turn causes BDNF downregulation [16]. Because the innervating neurons do not receive the neurotrophic factors their target counterpart generates, these two processes may result in their death [19]. A late-stage event during AD disease may be related to reduced BDNF levels [17]. Therefore, it is currently impossible to discern any discernible changes in serum BDNF levels in patients with mild cognitive impairment (MCI) [17]. A meta-analysis was carried out in 2019 by T. K. Ng et al. to compare the total serum BDNF levels between healthy controls and patients with AD and MCI [17]. Patients with AD had considerably reduced serum BDNF levels; p = 0.030, but no discernible changes were observed in the blood BDNF levels between MCI patients and healthy controls; p = 0.526 [17]. Overall, blood BDNF levels in AD patients are considerably lower than in healthy controls but still not in MCI patients [17]. In people with MCI, BDNF levels may be influenced by a complex interplay of biological characteristics, lifestyle choices, and psychosocial variables [17]. Ultimately, it could be the overabundance of amyloid beta (Aβ) or the inability to eliminate it that sets off synaptic malfunction and the unstoppable neurodegenerative process [20].

The build-up of amyloid beta leads to increased phosphorylation and secretion of Tau, an axonal protein associated with microtubules highly expressed in cortical neurons [19]. Neurodegeneration stems from disruptions in Tau metabolism, leading to the formation of neuritic plaques (NP)and hyperphosphorylated neurofibrillary tangles (NFTs) inside neurons [19]. BDNF deficiency is somehow linked to Aβ accumulation, Tau phosphorylation, neuroinflammation, and neuronal death [23].AD neuropathology is influenced by TrkB dysregulation and BDNF, particularly in the form of hippocampus NPs and NFTs [23]. According to these findings, NPs and NFTs can be prevented by attenuating BDNF/TrkB signaling deficiencies at the BDNF, TrkB, or downstream of TrkB signaling [23]. To evaluate the relationships between BDNF and TrkB expression and formation of NPs and NFTs within a homogeneous population of cyclic adenosine monophosphate (CA1) pyramidal neurons and regional hippocampal dissections utilizing postmortem tissues, S.D. Ginsberg et al. undertook a single-population longitudinal cohort study [23]. Gene expression data collected from a single population of CA1 pyramidal neurons and regional hippocampal dissections from Rush Religious Orders Study (RROS) participants were used to run negative binomial (NB) regressions [23]. As AD progresses, the downregulation of BDNF and TrkB is linked independently to the elevated levels of entorhinal cortex NFTs and CA1 NPs [23]. There is likely more than one way for BDNF signaling to influence the cascades that result in the creation of plaque and tangles; the exact mechanism is still unknown [23]. Table 4 summarizes the research on the effects of altered BDNF levels in AD patients and the relationship between BDNF/TrkB downregulation leading to AD development.

**Table 8:** Effects of altered BDNF levels in AD patients BDNF, brain-derived neurotrophic factor; AD, Alzheimer’s disease; MCI, mild cognitive impairment; NPs, neuritic plaques; NFTs, neurofibrillary tangles

Author	Study Type	Purpose of Study	Conclusion
T. Siang Ng et al. [17]	Systematic review and Meta analysis	To assess the serum levels of BDNF in AD patients and individuals with MCI	Serum BDNF levels were significantly lower in patients with AD, no significant difference was observed when comparing the serum BDNF levels between individuals with MCI and healthy controls
S.D. Ginsberg, et al. [23]	Longitudinal Cohort Study	To assess the association of BDNF/TrkB in the formation of NPs and NFTs within a homogenous population of CA1 pyramidal neurons	Downregulation of BDNF and TrkB is independently associated with increased entorhinal cortex NFTs and CA1 NPs during the progression of AD

Hence, by addressing mechanisms of neuroplasticity and avoiding or attenuating amyloid and tau pathology, restoration of BDNF/TrkB signaling, whether through neurotrophins administration, transactivation of the TrkB receptor, or activation of downstream pathways, may provide neuroprotection [2].

Role of Neurotrophins in Parkinson's Disease

As of right now, Parkinson’s disease (PD) is the second most prevalent neurodegenerative illness globally [24]. Dopaminergic neurons in the substantia nigra die out due to progressive neurodegeneration [15,24]. A considerable body of data suggests multifactorial etiology, including genetic and environmental factors [15,18,24]. Four pathognomonic markers are associated with PD: abnormal aggregation of the alpha-synuclein protein, neurodegeneration of the nigrostriatal system, neuroinflammation and oxidative stress, and motor and non-motor deficits [18,25]. Lewy body deposition and dopaminergic neuronal degeneration in the substantia nigra pars compacta are the key pathognomonic features of brain tissue examined in Parkinson's disease patients [8,18]. Unusual protein aggregates called Lewy bodies are found intracytoplasmically in neurons [18]. They are identified as eosinophilic masses that might be single, numerous, spherical, or elongated, leading to marked degeneration of dopaminergic neurons, neuron axons that carry afferent axons to the striatum in PD [18]. This results in changes to striatal morphology, such as modified synaptic connections and reduced spine density, and eventually leads to the loss of DA neurons in the midbrain, which causes symptoms that are both motor and non-motor [18]. PD is typified by motor symptoms, which appear after dopamine neuron loss and degeneration reach at least 30%, but other estimates put that number as high as 60% [18]. This may be a window of opportunity where therapies, even if they only slightly slow down the rate of neurodegeneration, could significantly affect how well a patient responds to treatment [18]. Signs of persistent neuroinflammation, such as reactive astrocyte and microglia markers, are another pathological characteristic of Parkinson's disease patients' brains [18]. The pathophysiology involves the accumulation of Lewy bodies and related oxidative stress, mitochondrial malfunction, apoptosis, and cytokine release [14,18]. The neurotrophin known as brain-derived neurotrophic factor (BDNF) stimulates arborization, synaptic plasticity, dendritic morphogenesis, and even neurogenesis in adult brains [14,26]. The growth of dopaminergic neurons of the substantia nigra, which are broadly distributed in cortical and subcortical regions, depends on brain-derived neurotrophic factors [14,26]. Consequently, mature dopaminergic neurons die when BDNF expression is blocked [14,26]. The leading players in the neuropathology of PD are BDNF and its receptor, TrkB [14]. A deficiency in the TrkB receptor leads to the accumulation of alpha-synuclein in the substantia nigra and the degeneration of dopaminergic neurons. Additionally, early-stage PD neuropathology is characterized by decreased BDNF/TrkB signaling [14]. At glutamatergic synapses, BDNF-TrkB signaling increases the number of docked vesicles within active zones [18]. It also modifies the activation kinetics of inhibitory gamma-aminobutyric acid (GABA) and N-methyl-D-aspartate (NMDA) receptors in the postsynaptic membrane [18]. Interestingly, a higher frequency of familial Parkinson's disease has been associated with BDNF polymorphism [14].

The pathophysiology of PD and the loss of nigral dopaminergic neurons may be related to the reduction in brain dopaminergic neurons in the substantia nigra pars compacta (SNpc) of PD patients, as there is a lower expression of BDNF in these neurons [15]. Strong evidence currently exists connecting BDNF to Parkinson's disease. For example, several studies showed that individuals with Parkinson's disease had significantly lower serum levels of BDNF [24,26]. Using a sample of 1496 participants (838 PD patients and 657 healthy controls), L. Jiang et al. conducted a meta-analysis to investigate the correlation between BDNF levels in PD patients and healthy controls [24]. They found that PD patients had significantly lower serum levels of BDNF than the healthy controls (SMD= -1.03; 95% CI [-1.83, -0.23]; P=.012) [24]. Z. Chen et al. conducted an additional analysis to examine the amount of BDNF in people with PD [26]. The study comprised 1920 participants, of which 1034 had Parkinson's disease, and 886 were healthy controls. Subjects with both PD and depression, as well as PD alone, had significantly decreased BDNF levels when compared to healthy controls. Nevertheless, there was no appreciable variation in BDNF levels between those with PD and depression and those without PD and depression. Table 5 provides a detailed display of these meta-analyses.

**Table 9:** Meta-analyses studies explaining the effects of altered serum BDNF levels in PD patients BDNF, Brain-derived neurotrophic factor; PD, Parkinson’s disease

Author	Intervention Studied	Number of Patients	Study Type	Conclusion
L. Jiang et al. [24]	Serum BDNF levels	1496 participants (838 PD patients and 657 healthy controls)	Systematic review and meta-analysis	significantly decreased serum levels of BDNF in PD patients when compared with the healthy controls
Z. Chen et al. [26]	Serum BDNF levels	1920 subjects (1034 PD patients and 886 healthy controls)	Systematic review and meta-analysis	BDNF levels were significantly lower in the subjects with PD combined with depression, and PD without depression as compared to healthy controls

Major pro-survival and anti-apoptotic pathways, such as the MAPK and AKT pathways, appear to be activated by neurotrophins. If neurotrophins prevent apoptosis, neurons may live longer; nonetheless, the damage will accrue, and eventually, the neurons will die from non-apoptotic causes. However, extending the life of the surviving neurons would still be very advantageous [18]. Neurotrophins can expand the survival of the 40-60% dopamine cells that remain at the onset of motor symptoms if delivered soon after diagnosis [18]. This may help specific neurons that are still alive but have lost their dopamine phenotype to function again [18]. 

Limitations

This review used five databases spanning 2014 to 2024 and only included human studies published in English. Furthermore, only complete free-text publications were obtained, and animal studies were not included, which might have excluded some relevant research.

## Conclusions

The evidence currently available about the involvement of neurotrophins and their signaling pathways in the complex neuropathology that underlies the onset of Parkinson's and Alzheimer's diseases has been assessed in this study. As previously mentioned, studies on the crucial roles these neurotrophins play in neurogenesis and neuroplasticity might be influential in halting the progression of the disease. They may even reverse the course to some extent. To sum up, neurotrophins can be represented as intriguing targets for therapeutic intervention that may alter or even reverse the progression of neuropathology linked to neurodegenerative diseases. Given the complexity of the condition and the paucity of available data, more research is required to clarify the effectiveness of neurotrophins in preclinical and clinical studies, emphasizing their mode of action, adverse effects, and the most secure way to administer them to patients.
